# Frequency of color blindness in pre-employment screening in a tertiary health care center in Pakistan

**DOI:** 10.12669/pjms.332.11710

**Published:** 2017

**Authors:** Shaukat Ali Chhipa, Farzeen K. Hashmi, Shehreen Ali, Mustafa Kamal, Khabir Ahmad

**Affiliations:** 1 Dr. Shaukat Ali Chhipa, FCPS. Department of Surgery, Aga Khan University Hospital, Karachi, Pakistan; 2 Dr. Farzeen K. Hashmi, MBBS. Department of Surgery, Aga Khan University Hospital, Karachi, Pakistan; 3 Ms. Shehreen Ali, BSN. Department of Surgery, Aga Khan University Hospital, Karachi, Pakistan; 4 Dr. Mustafa Kamal, MBBS. Department of Surgery, Aga Khan University Hospital, Karachi, Pakistan; 5 Dr. Khabir Ahmad, PhD. Department of Surgery, Aga Khan University Hospital, Karachi, Pakistan

**Keywords:** Color vision deficiency, Pre-employment examination, Heath care

## Abstract

**Objective:**

To describe the frequency of color vision deficiency among Pakistani adults presenting for pre-employment health screening in a tertiary care hospital.

**Methods:**

The cross-sectional study was carried out at the Aga Khan University Hospital, Karachi, and the data was collected for color vision deficiency, age, gender, and job applied for from pre-employment examination during 2013-2014. IBM SPSS 20 was used for statistical analysis.

**Results:**

Three thousand four hundred and thirty seven persons underwent pre-employment screening during 2013 and 2014; 1837 (53.44%) were males and 1600 (46.65%) females. The mean age was 29.01 (±6.53) years. A total of 0.9% (32/3437) persons had color vision deficiency with male being 1.4% and female 0.4%.

**Conclusion:**

Color vision deficiency was observed in 0.9% of candidates screened for pre-employment health check up in a tertiary care hospital. The color vision deficiency was predominantly present in male individuals.

## INTRODUCTION

Color blindness is the inability to perceive color differences under normal lighting conditions. It is most commonly inherited from mutations on X chromosome and thus, more common in men than women. Prevalence of deficiency in European Caucasians is about 8% in men and about 0.4% in women and between 4% and 6.5% in men of Chinese and Japanese ethnicity.^1^ However, the male: female prevalence ratio is markedly different in Europeans and Asians.^1^

Few prevalence studies have been reported from other parts of the world such as Turkey (7.3%), Iran (4.7%), India (2.8% to 8.2%, ethnic variations), Saudi Arabia (2.9%).^2^ However, Siddiqui compared the medical and non-medical students of Pakistan and found 2.75% overall prevalence for color vision deficiency (CVD)^3^ and Hamida reported overall 2.48% CVD in population of Quetta, Pakistan.^4^ Although color blindness does not cause any significant disability, it does keep one from performing certain jobs or causes hindrance in some ways. There is dearth of information on the impact that color vision deficiency has on employment opportunities in adults. Color vision standards are established in aviation and railway fields; however for drivers of motor vehicles they have not been effectively adopted. Similarly for health professionals’ specific standards have not been set.

We are observing an increasing number of pre-employment examination cases being referred from general physicians to the ophthalmology outpatient clinic for assessment of color vision deficit and medical fitness. The objective of our study was to assess the prevalence of color vision impairment among Pakistani adults presenting for pre-employment health screening in a tertiary care hospital.

## METHODS

This was a cross-sectional study. Ethical approval for this study was obtained from the Ethical Review Committee (ERC) of the Aga Khan University, Karachi, Pakistan. The study included all individuals who had applied for jobs in a single health care institution and had undergone pre-employment eye examination during 2013-2014. The exclusion criteria were to exclude the individuals who had history of central nervous system or anti-tuberculosis drugs and ocular or neurological surgeries. Fortunately no individual was fit on the criteria. It was non probability consecutive sampling. Our main outcome measure was color vision deficiency, which was determined using Ishihara color test. The Ishihara book of 38 plates was held parallel to the face at a distance of 75 cm from the candidate, perpendicular to the line of vision. Each plate was presented to the candidate for three to five seconds and they were requested to read the numbers. The persons who read all the plates properly were deliberated normal whereas the one who could not read the plates accurately was considered to be the color vision deficient. The categorization of color vision defects was studied with the aid of the key postulated with the chart. This is the most commonly used screening test for color deficiency. Two research officers collected data on candidate’s age, gender, color vision deficiency and job applied for using a structured proforma. Data were entered and analyzed using IBM SPSS Statistics version 20. Qualitative variables were reported as counts and percentages and quantitative variables as means and standard deviation. Chi-square tests were used to assess gender differences in color vision deficiency. A p-value of < 0.05 was considered statistically significant.

## RESULTS

A total of 3437 persons underwent pre-employment screening during 2013 and 2014. Out of these, 1837 (53.4%) were males and 1600 (46.6%) females. The mean age of candidates was 29.01 (±6.53) years. Overall, 0.9% (32/3437) persons had color vision deficiency (Table-I). Color blindness was present in 1 in 71 men and 1 in 266.6 women (1.41% vs. 0.37%; p = 0.002). Out of 32 persons found to have color vision deficiency, 18 (56.3%) had applied for nursing-related jobs, and 4 (12.5%) as junior doctors. Another 4 (12.5%) had applied in other high need area and 6 (18.8%) in low need areas.

**Table-I:** Frequency of colour blindness by gender and age group.

*Age group, years*	*Screened*	*Individuals with color vision deficiency*	

		*Frequency*	*%*
≤ 24	734	7	0.95
≥ 25	2703	25	0.92
**Total**	3437	32	0.93
***Sex***			
Male	1837	26	1.41
Female	1600	6	0.37
***Year***			
2013	1731	18	1.03
2014	1706	14	0.82

## DISCUSSION

Color vision deficiency can be difficult to detect. Pre-employment eye screening is important avenue to identify individuals with such deficiencies. In our study we determine the prevalence of color blindness among candidates for different positions in a large health care institution. The overall prevalence of color vision deficiency was 0.93%. Men were 3.8 times more likely to be color blind than women. Unanimously CVD is more prevalent in males as compared to females.^5^ The observation about females in our study was 0.37% which is comparable with large surveys that reported CVD in females of Greenland 0.4%, Ethiopia 0.2%, Iran 0.43%, Jordan 0.33% while lesser with females of Iraq 3.2%, Spain 0.75%, Saudi Arabia 0.75% and Denmark 0.54%.^6^,^7^ Instead we detected 1.41% males have CVD in our studied population, while internationally reported CVD in males are India 8.73%, Belgium 8%, United States 8%, Turkey 7.33% and China 6.5%. 7-9 Internationally reported overall CVD differs in different races and geographical areas.^7^,^10^

**Table-II:** Jobs that individuals with color vision deficiency had applied for.

*Job*	*Frequency*	*Percent*
Nursing^1^	18	56.25
Junior doctors^2^	4	12.50
Other high need areas^3^	4	12.50
Low need areas^4^	6	18.75

Total	32	100.0

1. Registered Nurse, Cardiac Perfusionist, Nursing Assistant, Nursing Intern, Patient Care Attendant.

2. Intern and Medical Officer.

3. Phlebotomist, Engineer, Trainee Pharmacist.

4. Field Worker, Research Associate, Marketing Executive, Trainee Physiotherapist, Unit Receptionist.

Health care workers with color deficiencies experience difficulties in identifying changes in body color such as pallor, cyanosis, jaundice and erythema.^11^ They also have difficulty in performing ophthalmic and otoscopic examinations or reading blood and urine test strips.^12^,^13^ Campbell determined that health care providers having color deficiencies were in difficulty to identify physical signs.^14^ This is compromising the patients’ safety. These difficulties are under-reported due to lack of screening before selecting or starting the profession in health care.

The ability to clearly differentiate color is essential for working in clinical laboratories. A study from Iran found that color vision deficient medical laboratory technicians can end up making a wide range of errors in lab tests and should not be considered medically fit for such employment choices.^15^

Color vision deficiency, though not very rare, remains an unnoticed problem most of the time. It has been reported that 96% of the color-blind students attending middle school and 65% of the colorblind university students are not aware of their anomalous vision status.^16^ Therefore knowing of their deficiency at a much earlier age will allow them to adapt a profession with low need of color vision.

## CONCLUSION

Color vision deficiency was observed in 0.9% of candidates who applied for the job in a tertiary care hospital. The observed CVD with convincing gender differentiation is in affirmation with global finding of male predominance. The affected candidates were screened out in pre-employment examination, therefore early screening during the school years would greatly help affected individuals in choosing their future professions.

### Authors’ Contribution

***SAC:*** Conceived, designed and did statistical analysis & editing of manuscript.

***FKH, SA and MK:*** Did data collection and manuscript writing.

***KA:*** Did review and final approval of manuscript.
